# Effect of regional medical disparities on complications in patients with hypertension: Cox’s proportional hazard models

**DOI:** 10.3389/fmed.2023.1138017

**Published:** 2023-06-02

**Authors:** Choa Yun, Minah Park, Jae Hong Joo, Soo Hyun Kang, Sung Hoon Jeong, Chung-Mo Nam, Eun-Cheol Park, Sung-In Jang

**Affiliations:** ^1^Department of Biostatistics and Computing, College of Medicine, Yonsei University, Seoul, Republic of Korea; ^2^Institute of Health Services Research, Yonsei University, Seoul, Republic of Korea; ^3^Department of Preventive Medicine, Eulji University School of Medicine, Daejeon, Republic of Korea; ^4^Department of Health Informatics and Biostatistics, Yonsei University Graduate School of Public Health, Seoul, Republic of Korea; ^5^Department of Preventive Medicine, Gachon University College of Medicine, Incheon, Republic of Korea; ^6^Department of Rehabilitation Medicine, Seoul National University Hospital, Seoul, Republic of Korea; ^7^Department of Preventive Medicine, Yonsei University College of Medicine, Seoul, Republic of Korea

**Keywords:** hypertension, adverse effect, healthcare disparity, complications, Cox proportional hazards models

## Abstract

**Objective:**

Complications associated with hypertension can be alleviated by providing necessary medical services. However, there may be disparities in their provision depending on regional differences. Thus, this study aimed to examine the effects of regional healthcare disparities on complications in patients with hypertension in South Korea.

**Methods:**

Data from the National Health Insurance Service National Sample Cohort (2004–2019) were analyzed. The position value for the relative composite index was used to identify medically vulnerable regions. The diagnosis of hypertension within the region was also considered. The risk of complications associated with hypertension included cardiovascular, cerebrovascular, and kidney diseases. Cox proportional hazards models were used for statistical analysis.

**Results:**

A total of 246,490 patients were included in this study. Patients who lived in medically vulnerable regions and were diagnosed outside their residential area had a higher risk of complications than those living in non-vulnerable regions and were diagnosed outside the residential area (hazard ratio: 1.156, 95% confidence interval: 1.119–1.195).

**Conclusion:**

Patients living in medically vulnerable regions who were diagnosed outside their residential areas were more likely to have hypertension complications regardless of the type of complication. Necessary policies should be implemented to reduce regional healthcare disparities.

## 1. Introduction

The prevalence of hypertension is high worldwide. If not treated properly, hypertension can lead to complications, including cardiovascular disease (1), cerebrovascular disease, and kidney disease (2). However, if well managed, disabilities and premature death can be prevented (3). In 2010, the worldwide prevalence of hypertension was estimated to be 1.39 billion persons, representing 31% of all adults (4). Since South Korea is becoming the fastest-aging country worldwide3, there is a greater likelihood of an increase in the prevalence of hypertension. According to the Korea Hypertension Fact Sheet 2021, among the adult population (aged ≥20 years), it was estimated that 28% have hypertension. Compared with that in 2002, the number of people diagnosed with hypertension increased from 3.0 million to 10.1 million in 2019 (5).

Despite the seriousness of hypertension, it can be treated with proper and effective management (6). Especially among patients with chronic diseases, continuity of care is known to result in better patient satisfaction and outcomes (7). However, managing chronic diseases becomes much more difficult for people residing in rural areas (8) because the population composition, socioeconomic conditions, and distribution of medical facilities are different and the use of medical care varies (9). As of 2022, Seoul, the capital of South Korea, had 4.8 doctors per 1,000 inhabitants, and except for metropolitan cities (Busan, Daegu), all the other areas had an average of less than 3.2 doctors per 1,000 inhabitants (10). In addition, in a study on amenable deaths for specific districts conducted in 2018, the death rate in Seochogu (a district in Seoul) was 25.8 per 100,000 people, whereas the death rate in Hapcheon-gun (a county in Gyeongsangnam-do) was 112.2 per 100,000 people, a 4.3 fold difference (11).

Considering that hypertension requires ongoing treatment, a process of identifying medical gaps by region is necessary to avoid potential complications. Various approaches have been taken to assess the healthcare gap according to the region. For example, a study of all-cause mortality among patients with hypertension was conducted based on the years of life lost according to region (11). Another study assessed disparities according to socioeconomic status (12). However, the results of these studies were reported by region without considering other factors; therefore, there is a need to assess the overall effect of healthcare factors on complications of hypertension.

Therefore, our study aimed to estimate the effect of regional healthcare disparities using the position value for relative composite (PARC) index on complications and morbidity events among patients with hypertension in the Korean population using data from the Korean Health Insurance Service.

## 2. Materials and methods

### 2.1. Data and study population

The data used in this study were obtained from the National Health Insurance Service National Sample Cohort (NHIS-NSC) (13) from 2002 to 2019. However, to prevent the effects of other comorbid conditions, the period 2002–2003 was designated as a washout period. In addition, to identify patients with newly diagnosed hypertension, those who were diagnosed with hypertension in 2002–2003 and those diagnosed with hypertension complications in 2002–2003 were excluded. Finally, the data from 2004 to 2019 were used. After these exclusions, the total study population included 246,490 individuals.

### 2.2. Ethical consideration

This study was reviewed and approved by the International Review Board of Yonsei University’s Health System (number: Y-2020-0031) and adheres to the tenets of the Declaration of Helsinki. The NHIS-NSC data do not contain any identifying information; hence, additional approval for written informed consent was not required.

### 2.3. Variables

The variables of interest in this study were medically vulnerable regions and diagnosis of hypertension within these regions. The medically vulnerable regions were identified using PARC indicators. These indicators have enabled the identification of healthcare levels by region in Korea (14). Details regarding analytical methods are provided in past literature (15–20).

PARC is an objective indicator that can identify relative locations compared with other regions using the positioning method. The PARC value ranges from −1 to 1. If the value is 1, it indicates superior healthcare levels in the region; 0 indicates average healthcare levels; and −1 indicates worst healthcare levels when compared with the mean value of the entire region. In other words, the closer the value is to-1, the lower the level of healthcare in the region than the average level of healthcare in the entire region, while the closer the value is to 1, the higher the average healthcare level of the entire region. In this study, when the PARC value was less than −0.33, it was classified as a medically vulnerable region (14).

The primary dependent variable in this study was hypertension complications among hypertension patients (International Classification of Diseases 10th revision codes: I10, I11, I12, I13, and I15). The complications included cardiovascular disease (ICD-10: I20, I21, I46, I48, and I50), cerebrovascular disease (ICD-10: I60, I61, I62, I63, and G45), and chronic kidney disease (ICD-10: N18.5).

The other covariates included sex (male/female), age (under 10, 10–19, 20–29, 30–39, 40–49, 50–59, 60–69, and over 70 years), type of insurance coverage (NHI/medical aid), income (low/middle/high), Charlson’s comorbidity index (CCI) (0, 1, 2+), and types of healthcare facility (tertiary hospital/primary care hospital).

### 2.4. Statistical analysis

Continuous variables are reported as means and standard deviations and were compared using Student’s t-test or analysis of variance for multiple groups, as appropriate. Categorical variables are reported as counts and percentages and were compared using the *χ*^2^ test. The primary analysis tested whether a statistical interaction was present between the residential region and diagnosis area regarding the event of the complication survival, which was defined as the time from diagnosis of essential hypertension to the time complications occurred. Cox proportional hazards models were fitted to our results, and adjusted estimations were reported. In the presence of an interaction effect (residential region × diagnosis area), statistical significance was considered at *p* < 0.05. After considering the level of each interaction term as one group and dividing it into four groups—vulnerable and outside region, vulnerable and inside region, non-vulnerable and outside region, and non-vulnerable and inside region—univariate and multivariate Cox proportional hazards model analyses were performed. Additionally, Cox proportional hazards models for subgroups were used to calculate the hazard ratio (HR) for complication events, including cardiovascular, kidney, and cerebrovascular diseases, with adjustments for potential confounders. All statistical analyses were performed using SAS version 7.1 Enterprise (SAS Institute, Cary, NC), and all *p*-values were two-sided with a significance level of 0.05.

## 3. Results

The study cohort comprised 246,490 participants (Table 1). Table 1 shows the descriptive statistics of baseline characteristics by group. Patients who lived in vulnerable regions and were diagnosed outside the regions comprised 4.47% (11,007) of the participants, and those from non-vulnerable regions who were diagnosed inside the region comprised 49.05% (120,893).

**Table 1:** Baseline characteristics by group (residential region × diagnosis area).

	Group				*p*-value
	Vulnerable and outside region (*n* = 11,007, 4.47%)	Vulnerable and inside region (*n* = 19,760, 8.02%)	Non-vulnerable and outside region (*n* = 94,830, 38.47%)	Non-vulnerable and inside region (*n* = 120,893, 49.05%)	
	*n* (%)	*n* (%)	*n* (%)	*n* (%)	
*Sex*					<0.0001
Male	5,512 (4.38)	8,721 (6.94)	52,253 (41.56)	59,232 (47.11)	
Female	5,495 (4.55)	11,039 (9.14)	42,577 (35.25)	61,661 (51.00)	
*Income*					
Low	1,895 (4.73)	3,324 (8.29)	14,613 (36.45)	20,261 (50.54)	<0.0001
Middle	4,854 (4.56)	9,170 (8.61)	39,654 (37.22)	52,857 (49.61)	
High	4,258 (4.26)	7,266 (7.28)	40,563 (40.62)	47,775 (47.84)	
*Coverage type*					<0.0001
NHI, employed/self-employed	4,779 (3.91)	7,919 (6.48)	50,383 (41.21)	59,171 (48.40)	
Medical aid	6,228 (5.01)	11,841 (9.53)	44,447 (35.78)	61,722 (49.68)	
*Age (years)*					<0.0001
Under 10	166 (25.78)	353 (12.30)	1,002 (34.90)	1,350 (47.02)	
10–19	83 (6.65)	52 (4.16)	527 (42.19)	587 (47.00)	
20–29	262 (5.36)	118 (2.41)	2,648 (54.18)	1859 (38.04)	
30–39	630 (3.51)	618 (3.44)	8,812 (49.12)	7,880 (43.92)	
40–49	1709 (3.30)	2,622 (5.06)	21,166 (40.83)	26,342 (50.82)	
50–59	2,674 (3.86)	4,631 (6.69)	20,833 (35.00)	29,701 (49.90)	
60–69	2,962 (4.98)	6,024 (10.12)	20,833 (35.00)	29,701 (49.90)	
Over 70	2,521 (6.47)	5,342 (13.72)	13,172 (33.83)	17,902 (45.98)	
*Charlson comorbidity index*					<0.0001
0	4,371 (4.50)	6,981 (7.19)	40,444 (41.67)	45,270 (46.64)	
1	3,312 (1.34)	6,283 (8.29)	28,501 (37.60)	37,709 (49.74)	
Over 2	3,324 (4.52)	6,496 (8.82)	25,885 (35.16)	37,914 (51.50)	
*Healthcare facility*					<0.0001
Tertiary Hospital	4,953 (7.56)	2,751 (4.20)	35,293 (53.89)	22,499 (34.35)	
Primary Care Hospital	6,054 (3.34)	17,009 (9.40)	59,537 (32.89)	98,394 (54.36)	

We fitted multivariate Cox proportional hazards models to examine the presence of an interaction effect. In this model, the interaction term between the residential region (vulnerable vs. non-vulnerable) and the diagnosis area (outside vs. inside) was significant in the adjusted models (*p* for interaction = 0.005) (Supplementary Table S1). Since the interaction term was significant, we compared HRs for our outcome in the four groups defined in the methods using univariate and multivariate Cox proportional hazards models. Table 2 shows that the vulnerable and outside region group had the highest rate of complications, and there was a significant association between patients who were included in this group and the risk of complications when compared with that in the reference group (unadjusted HR for complications, 1.465; 95% CI, 1.418–1.513; *p* < 0.0001 and adjusted HR for complications, 1.156; 95% CI, 1.119–1.195; *p* < 0.0001).

**Table 2:** Cox proportional hazard regression models of time to complication event.

	Univariate		Multivariate	
	Hazard ratio (95% CI)	*p*-value	Hazard ratio (95% CI)	*p*-value
Group				
Vulnerable and Outside region	1.465 (1.418, 1.513)	<0.0001	1.156 (1.119, 1.195)	<0.0001
Vulnerable and Inside region	1.146 (1.116, 1.177)	<0.0001	1.045 (1.017, 1.073)	0.0015
Non-vulnerable and Outside region	1.149 (1.131, 1.167)	<0.0001	1.042 (1.025, 1.059)	<0.0001
Non-vulnerable and Inside region	Ref		Ref	
Sex				
Male	1.3001 (0.986, 1.015)	0.9184	1.158 (1.139, 1.177)	<0.0001
Female	Ref		Ref	
Income				
Low	0.981 (0.960, 1.003)	0.0876	1.036 (1.013, 1.059)	0.0017
Middle	0.936 (0.922, 0.951)	<0.0001	1.021 (1.005, 1.037)	0.0113
High	Ref		Ref	
Coverage type				
NHI, employed/ self-employed	0.847 (0.834, 0.859)	<0.0001	0.954 (0.937, 0.970)	<0.0001
Medical aid	Ref		Ref	
Age (years)				
Under 10	1.310 (1.234, 1.390)	<0.0001	1.435 (1.352, 1.523)	0.0001
10–19	0.094 (0.072, 1.121)	<0.0001	0.092 (0.071, 0.119)	<0.0001
20–29	0.186 (0.170, 0.204)	<0.0001	0.183 (0.166, 0.200)	<0.0001
30–39	0.297 (0.285, 0.309)	<0.0001	0.302 (0.290, 0.315)	<0.0001
40–49	0.403 (0.394, 0.413)	<0.0001	0.413 (0.402, 0.423)	<0.0001
50–59	0.535 (0.524, 0.547)	<0.0001	0.535 (0.524, 0.547)	<0.0001
60–69	0.722 (0.707, 0.737)	<0.0001	0.714 (0.700, 0.729)	<0.0001
Over 70	Ref		Ref	
Charlson comorbidity index				
0	0.623 (0.612, 0.635)	<0.0001	0.667 (0.655, 0.679)	<0.0001
1	0.806 (0.792, 0.820)	<0.0001	0.824 (0.810, 0.838)	<0.0001
Over 2	Ref		Ref	
Healthcare facility				
Tertiary Hospital	1.646 (1.621, 1.671)	<0.0001	1.707 (1.680, 1.734)	<0.0001
Primary Care Hospital	Ref		Ref	

The results of the *χ*^2^ test indicated that each complication rate was significantly different among the four groups (*p* < 0.0001) (Table 3). In addition, on examining the subgroups, it was observed that patients diagnosed outside their residential area and those living in medically vulnerable areas had the highest rate of all complications (cardiovascular disease: 2640 [24.0%]; kidney disease: 262 [2.4%]; cerebrovascular disease: 1928 [17.5%]).

**Table 3:** General characteristics of study subjects for hypertension complications.

	Hypertension complications											
	Complications		*p*-value	Cardiovascular disease		*p*-value	Kidney disease		*p*-value	Cerebrovascular disease		*p*-value
	Yes	No		Yes	No		Yes	No		Yes	No	
	*n* (%)	*n* (%)		*n* (%)	*n* (%)		*n* (%)	*n* (%)		*n* (%)	*n* (%)	
*Group*			<0.0001			<0.0001			<0.0001			<0.0001
Vulnerable and Outside region	4,100 (37.3)	6,907 (62.8)		2,640 (24.0)	8,367 (76.0)		262 (2.4)	10,745 (97.6)		1928 (17.5)	9,079 (82.5)	
Vulnerable and Inside region	6,341 (32.1)	13,419 (67.9)		3,791 (19.2)	15,969 (80.8)		326 (1.7)	19,434 (98.3)		3,330 (16.9)	16,430 (83.1)	
Non-vulnerable and Outside region	28,353 (29.9)	66,477 (70.1)		18,768 (19.8)	76,062 (80.2)		2,123 (2.2)	92,707 (97.8)		12,114 (12.8)	82,716 (87.2)	
Non-vulnerable and Inside region	33,846 (28.0)	87,047 (72.0)		21,533 (17.8)	99,360 (82.2)		2,184 (1.8)	118,709 (98.2)		15,520 (12.8)	105,373 (87.2)	
*Sex*			<0.0001			<0.0001						<0.0001
Male	35,033 (27.9)	90,685 (72.1)		22,562 (18.0)	103,156 (82.0)		2,957 (2.4)	122,761 (97.6)		15,064 (12.0)	110,654 (88.0)	
Female	37,607 (31.1)	83,165 (68.9)		24,170 (20.0)	96,602 (80.0)		1938 (1.6)	118,834 (98.4)		17,828 (14.8)	102,944 (85.2)	
*Income*			<0.0001			<0.0001			0.2322			<0.0001
Low	11,316 (28.2)	28,777 (71.8)		7,140 (17.8)	32,953 (82.2)		757 (1.9)	39,336 (98.1)		5,204 (13.0)	34,889 (87.0)	
Middle	30,428 (28.6)	76,107 (71.4)		19,257 (18.1)	87,278 (81.9)		2,112 (2.0)	104,423 (98.0)		13,981 (13.1)	92,554 (86.9)	
High	30,896 (30.9)	68,966 (69.1)		20,335 (20.4)	79,527 (79.6)		2026 (2.0)	97,836 (98.0)		13,707 (13.7)	86,155 (86.3)	
*Coverage type*			<0.0001			<0.0001			0.0356			<0.0001
NHI, employed/ self-employed	31,940 (26.1)	90,312 (73.9)		20,970 (17.2)	101,282 (82.9)		2,355 (1.9)	119,897 (98.1)		13,429 (11.0)	108,823 (89.0)	
Medical aid	40,700 (32.8)	83,538 (67.2)		25,762 (20.7)	984,765 (79.3)		2,540 (2.0)	121,698 (98.0)		19,463 (15.7)	104,775 (84.3)	
*Age (years)*			<0.0001			<0.0001			<0.0001			<0.0001
Under 10	1,159 (40.4)	1712 (59.6)		707 (24.6)	2,164 (75.4)		36 (1.3)	2,835 (98.7)		613 (21.4)	2,258 (78.6)	
10–19	58 (4.64)	1,191 (95.4)		45 (3.6)	1,204 (96.4)		7 (0.6)	1,242 (99.4)		7 (0.6)	1,242 (99.4)	
20–29	464 (9.49)	4,423 (90.5)		322 (6.6)	4,565 (93.4)		94 (1.9)	4,793 (98.1)		75 (1.5)	4,812 (98.5)	
30–39	2,724 (15.18)	15,216 (84.8)		1859 (10.4)	16,081 (89.6)		325 (1.8)	17,615 (98.2)		773 (95.7)	17,167 (4.3)	
40–49	10,815 (20.86)	41,024 (79.1)		7,245 (14.0)	44,594 (86.0)		762 (1.5)	51,077 (98.5)		4,066 (92.2)	47,773 (7.8)	
50–59	18,910 (27.31)	50,337 (72.7)		12,510 (18.1)	56,737 (81.9)		1,148 (1.7)	68,099 (98.3)		8,025 (88.4)	61,222 (11.6)	
60–69	22,621 (38.0)	36,899 (62.0)		14,452 (24.3)	45,086 (75.7)		1,592 (2.7)	57,928 (97.3)		10,995 (81.5)	48,525 (18.5)	
Over 70	15,889 (40.8)	23,048 (59.2)		3,592 (24.6)	29,345 (75.4)		931 (2.4)	38,006 (97.6)		8,338 (78.6)	30,599 (21.4)	
*Charlson comorbidity index*			<0.0001			<0.0001			<0.0001			<0.0001
0	17,424 (18.0)	79,642 (82.0)		10,626 (11.0)	86,440 (89.0)		1,129 (1.2)	95,937 (98.8)		7,405 (7.6)	89,661 (92.4)	
1	22,262 (29.4)	53,543 (70.6)		14,128 (18.6)	61,677 (81.4)		1,486 (2.0)	74,319 (98.0)		9,838 (13.0)	65,967 (87.0)	
Over 2	32,954 (44.8)	40,665 (55.2)		21,978 (29.9)	51,641 (70.2)		2,280 (3.1)	71,339 (96.9)		15,649 (21.3)	57,970 (78.7)	
*Healthcare facility*			<0.0001			<0.0001			<0.0001			<0.0001
Tertiary Hospital	25,980 (39.7)	39,516 (60.3)		17,411 (26.6)	48,085 (73.4)		1979 (3.0)	63,517 (97.0)		11,107 (17.0)	54,389 (83.0)	
Primary Care Hospital	46,660 (25.8)	134,334 (74.2)		29,321 (16.2)	151,673 (83.8)		2,916 (1.6)	178,078 (98.4)		21,785 (12.0)	159,209 (88.0)	

Table 4 shows the overall HR (95% CI) and *p*-value of the fitted multivariate Cox proportional hazards models with adjustments for potential confounders for the subgroup analysis. For cardiovascular and cerebrovascular diseases, the vulnerable and outside region group had the highest rate of complications when compared with the reference group (adjusted HR for cardiovascular disease, 1.167; 95% CI, 1.120–1.215; *p* < 0.0001 and adjusted HR for cerebrovascular disease, 1.162; 95% CI, 1.108–1.219; *p* < 0.0001). However, the non-vulnerable and outside region group had the highest rate of complications when compared with the reference group for kidney disease (adjusted HR, 1.124; 95% CI, 1.106–1.196; *p* < 0.0002). The HR for cardiovascular and kidney diseases was higher in the group with the first diagnosis outside the residential area, regardless of whether they were medically vulnerable. In contrast, the HR for cerebrovascular diseases was higher in the medically vulnerable group than in the diagnosis group.

**Table 4:** Cox proportional hazard regression models of time to complication event for specific hypertension complications.

	Cardiovascular disease		Kidney disease		Cerebrovascular disease	
	Hazard ratio (95% CI)	*p*-value	Hazard ratio (95% CI)	*p*-value	Hazard ratio (95% CI)	*p*-value
Group						
Vulnerable and Outside region	1.167 (1.120, 1.215)	<0.0001	1.081 (0.949, 1.231)	0.2413	1.162 (1.108, 1.219)	<0.0001
Vulnerable and Inside region	0.992 (0.958, 1.027)	0.6575	0.876 (0.780, 0.985)	0.0268	1.148 (1.105, 1.192)	<0.0001
Non-vulnerable and Outside region	1.080 (1.058, 1.102)	<0.0001	1.124 (1.056, 1.196)	0.0002	0.984 (0.960, 1.009)	0.2088
Non-vulnerable and Inside region	Ref		Ref		Ref	
Sex						
Male	1.061 (1.040, 1.083)	<0.0001	1.864 (1.749, 1.987)	<0.0001	1.065 (1.039, 1.090)	<0.0001
Female	Ref		Ref		Ref	
Income						
Low	0.931 (0.906, 0.957)	<0.0001	1.110 (1.020, 1.208)	0.0160	1.044 (1.010, 1.079)	0.0102
Middle	0.942 (0.923, 0.961)	<0.0001	1.071 (1.007, 1.139)	0.0288	1.062 (1.037, 1.087)	<0.0001
High	Ref		Ref		Ref	
Coverage type						
NHI, employed/self-employed	0.987 (0.966, 1.008)	0.2360	0.886 (0.830, 0.946)	0.0003	0.904 (0.882, 0.928)	<0.0001
Medical aid	Ref		Ref		Ref	
Age (years)						
Under 10	1.248 (1.156, 1.348)	<0.0001	0.781 (0.559, 1.089)	0.1454	1.202 (1.107, 1.305)	<0.0001
10–19	0.150 (0.112, 0.201)	<0.0001	0.196 (0.093, 0.413)	<0.0001	0.027 (0.013, 0.057)	<0.0001
20–29	0.263 (0.235, 0.294)	<0.0001	0.654 (0.527, 0.811)	0.0001	0.072 (0.057, 0.091)	<0.0001
30–39	0.398 (0.378, 0.419)	<0.0001	0.624 (0.548, 0.712)	<0.0001	0.196 (0.182, 0.211)	
40–49	0.521 (0.505, 0.538)	<0.0001	0.509 (0.461, 0.563)	<0.0001	0.344 (0.331, 0.358)	<0.0001
50–59	0.667 (0.649, 0.686)	<0.0001	0.562 (0.514, 0.614)	<0.0001	0.494 (0.479, 0.510)	<0.0001
60–69	0.866 (0.844, 0.889)	<0.0001	0.859 (0.791, 0.932)	0.0003	0.750 (0.729, 0.772)	<0.0001
Over 70	Ref		Ref		Ref	
Charlson comorbidity index						
0	0.492 (0.481, 0.504)	<0.0001	0.603 (0.561, 0.649)	<0.0001	0.519 (0.505, 0.534)	<0.0001
1	0.710 (0.695, 0.725)	<0.0001	0.799 (0.748, 0.853)	<0.0001	0.710 (0.692, 0.728)	<0.0001
Over 2	Ref		Ref		Ref	
Healthcare facility						
Tertiary Hospital	1.838 (1.802, 1.874)	<0.0001	1.755 (1.653, 1.864)	<0.0001	1.552 (1.515, 1.590)	<0.0001
Primary Care Hospital	Ref		Ref		Ref	

## 4. Discussion

In this study, we examined the effects of regional medical disparities on complications in patients with hypertension. Our results showed that patients living in medically vulnerable regions and those diagnosed outside their residential areas were more likely to have hypertension complications. In particular, for each individual complication, the results also showed that patients living in medically vulnerable regions and those diagnosed outside their residential areas were more likely to have a higher HR.

The results of this study are similar to those of studies conducted in other countries. For example, in China, which has a similar prevalence of hypertension to South Korea, treatment and control are worse in rural areas (21). In Romania, prevalence of hypertension is higher and general control of hypertension is lower in rural areas than in urban areas (22). In the United States, the prevalence of hypertension and current medication use is higher in rural areas due to lack of healthcare workers and a higher prevalence of obesity, lack of physical activity, and smoking (23).

The reason for the higher risk of hypertension complications may be disparities in access to care. With regard to hypertension management, counseling and education by health clinics and doctors are known to affect the recognition and treatment rate of hypertension (24). However, those in rural areas have limited access to healthcare personnel (25) and this problem is also present in Korea. While the number of doctors per 100 beds in metropolitan cities was 14.17 and the number of nurses was 60.95, in rural areas, the number of doctors was 6.36 and the number of nurses was 26.67 (26), which indicated a large gap in the access to healthcare personnel. In the case of departments, except for orthopedics, those in rural areas have limited access to other departments (27).

According to the subgroup analysis, patients living in medically vulnerable regions who were diagnosed outside their residential areas were more likely to have a higher risk of hypertension complications from cardiovascular, kidney, and cerebrovascular diseases. Although patient differences in characteristics, the overall prevalence could be attributed to medical access issues. Rural areas are known to have transportation issues and limited access to care, resulting in a higher likelihood of fewer hospital visits and under-diagnoses (28). As cardiovascular and cerebrovascular diseases are time-sensitive diseases with a “golden time” for the use of appropriate treatment (29, 30) implementation of timely and appropriate medical resources in both internal and external medical institutions is important (29). However, as patients in vulnerable regions and those who have been diagnosed outside their region have a significant shortage of these resources, there is a higher probability that they are more affected than other groups.

In the case of kidney disease, managing and controlling blood pressure is the best prevention method (31); therefore, accessibility and performance of primary care services are important (32). However, in South Korea, there is a preference for hospitals over clinics (33); thus, there has been a constant decrease in the number of clinics in rural areas over the past years (34). These factors may have had a greater effect on people diagnosed outside their region. This may indicate that a person’s most frequently used healthcare facility is outside their region, making accessibility much more difficult.

This study has some limitations. First, due to the use of medical claims data, factors such as obesity, physical activity, and smoking rate could not be considered. However, in South Korea, there are relatively small differences in the smoking rate (35), obesity (36) and amount of physical activity (37) between rural and urban areas; hence, it is unlikely that there is confounding by these factors. Nevertheless, these factors should be considered in future studies. Second, factors such as history of hypertension prescription and continuity of care could not be considered in this study. Future studies should consider these factors.

However, our study has some major strengths. To the best of our knowledge, this was the first study to investigate regional medical disparities in complication morbidity events in patients with hypertension using the PARC index. In addition, since all Korean citizens are required to enroll in the National Health Insurance Service, the NHIS datasets provide nationally representative data.

Our findings suggest that patients who live in medically vulnerable regions and are diagnosed outside their residential area are more likely to have a complication of hypertension, including cardiovascular and cerebrovascular disease. Therefore, necessary policies should be implemented to reduce regional healthcare disparities.

## Data availability statement

The datasets GENERATED/ANALYZED for this study can be found in the NHIS REPOSITORY https://nhiss.nhis.or.kr/bd/ab/bdaba000eng.do;jsessionid=65hUSEvUQ4RZuiKJOf9ggWNyYYMWwCUCBKUkhbpCMifjCNazbC9wHff1VfMi61cp.primrose22_servlet_engine10.

## Ethics statement

The studies involving human participants were reviewed and approved by International Review Board of Yonsei University’s Health System. Written informed consent for participation was not required for this study in accordance with the national legislation and the institutional requirements.

## Author contributions

CY and MP contributed to conception and design of the study. CY performed the statistical analysis. MP wrote the manuscript. All authors contributed to the article and approved the submitted version.

## Funding

This research was supported by a grant from the Korea Health Technology R&D Project through the Korea Health Industry Development Institute (KHIDI), funded by the Ministry of Health & Welfare, Republic of Korea (grant number: HI20C1130). Also, this work was also supported by the National Research Foundation of Korea (NRF; grant 2022R1F1A1062794) funded by the Korea government (Ministry of Science and ICT [information and communication technology]).

## Conflict of interest

The authors declare that the research was conducted in the absence of any commercial or financial relationships that could be construed as a potential conflict of interest.

## Publisher’s note

All claims expressed in this article are solely those of the authors and do not necessarily represent those of their affiliated organizations, or those of the publisher, the editors and the reviewers. Any product that may be evaluated in this article, or claim that may be made by its manufacturer, is not guaranteed or endorsed by the publisher.

## Supplementary material

The Supplementary material for this article can be found online at: https://www.frontiersin.org/articles/10.3389/fmed.2023.1138017/full#supplementary-material
