# Supplementary material for: Effect of regional medical disparities on complications in patients with hypertension: Cox’s proportional hazard models
Source: Front Med (Lausanne). 2023 Jun 2;10:1138017. doi: 10.3389/fmed.2023.1138017 (PMC10272513; doi:10.3389/fmed.2023.1138017)
Supplement: Supplementary file 1 [file Table_1.docx]

Supplementary Material

Effect of Regional Medical Disparities on Complications in Patients with Hypertension: Cox’s Proportional Hazard Models

Choa Yun^1†^, Minah Park^2†^, Jae Hong Joo^3^, Soo Hyun Kang^4^, Sung Hoon Jeong^5^, Chung-Mo Nam^6^, Eun-Cheol Park^2,6^, Sung-In Jang^2,6*^

*** Correspondence:**Sung-In Jang, MD, PhD

JANGSI@yuhs.ac

# Supplementary Table

# Supplementary Table S1 – Cox Proportional Hazard Regression Model of Time to Complication Event using interaction effects

|  | **Multivariate** | |
| --- | --- | --- |
|  | **Estimate (S.E)** | **P-value** |
| ***Region*** |  |  |
| **Vulnerable region** | 0.04 (0.01) | <.0001 |
| **Non-vulnerable region** | Ref |  |
| ***Diagnosis Area*** |  |  |
| **Outside the residential area** | 0.04 (0.01) | 0.0015 |
| **In the residential area** | Ref |  |
| ***Region***$\boldsymbol{\times}$***Diagnosis Area*** | 0.06 (0.02) | 0.0050 |
| ***Sex*** |  |  |
| **Male** | 0.15 (0.01) | <.0001 |
| **Female** | Ref |  |
| ***Income*** |  |  |
| **Low** | 0.04 (0.01) | 0.0017 |
| **Middle** | 0.02 (0.01) | 0.0113 |
| **High** | Ref |  |
| ***Coverage type*** |  |  |
| **NHI, employed/ self-employed** | -0.05 (0.01) | <.0001 |
| **Medical aid** | Ref |  |
| ***Age (years)*** |  |  |
| **Under 10** | 0.36 (0.03) | <.0001 |
| **10-19** | -2.39 (0.13) | <.0001 |
| **20-29** | -1.70 (0.05) | <.0001 |
| **30-39** | -1.20 (0.02) | <.0001 |
| **40-49** | -0.89 (0.01) | <.0001 |
| **50-59** | -0.62 (0.01) | <.0001 |
| **60-69** | -0.34 (0.01) | <.0001 |
| **Over 70** | Ref |  |
| ***Charlson comorbidity index*** |  |  |
| **0** | -0.41 (0.01) | <.0001 |
| **1** | -0.19 (0.01) | <.0001 |
| **Over 2** | Ref |  |
| ***Healthcare facility*** |  |  |
| **Tertiary Hospital** | 0.53 (0.01) | <.0001 |
| **Primary Care Hospital** | Ref |  |
